# Prognostic significance of total metabolic tumor volume on ^18^F-fluorodeoxyglucose positron emission tomography/ computed tomography in patients with diffuse large B-cell lymphoma receiving rituximab-containing chemotherapy

**DOI:** 10.18632/oncotarget.20447

**Published:** 2017-08-24

**Authors:** Chin-Chuan Chang, Shih-Feng Cho, Ya-Wen Chuang, Chia-Yang Lin, Shu-Min Chang, Wen-Ling Hsu, Ying-Fong Huang

**Affiliations:** ^1^ Department of Nuclear Medicine, Kaohsiung Medical University Hospital, Kaohsiung, Taiwan; ^2^ Institute of Clinical Medicine, Kaohsiung Medical University, Kaohsiung, Taiwan; ^3^ Division of Hematology & Oncology, Department of Internal Medicine, Kaohsiung Medical University Hospital, Kaohsiung, Taiwan; ^4^ Department of Medical Imaging and Radiological Sciences, Kaohsiung Medical University, Kaohsiung, Taiwan

**Keywords:** diffuse large B-cell lymphoma, MTV, TLG, FDG PET/CT, prognosis

## Abstract

**Purpose:**

The purpose of this study was to determine the prognostic significance of metabolic parameters on pre-treatment ^18^F-fluorodeoxyglucose positron emission tomography/ computed tomography (FDG PET/CT), in patients with diffuse large B-cell lymphoma (DLBCL) receiving rituximab-containing therapy.

**Materials and Methods:**

From September 2009 to December 2014, DLBCL patients who had received FDG PET/CT scans for staging were enrolled. The maximal standardized uptake value of tumor (SUVt) was recorded. The metabolic tumor volume (MTV) was the volume of lesion with an elevated SUV greater than 2.5. The total lesion glycolysis (TLG) was the sum of the products of MTV and mean SUV in all measured lesions. Univariate and multivariate analyses were used to assess the prognostic significance of maximal SUVt, total MTV, TLG and other clinical parameters.

**Results:**

There were 118 patients enrolled in this study. The median follow-up time was 28.7 months. The 5-year progression-free survival (PFS) for patients with higher and lower total MTV was 32.3% and 66.0% respectively (*p* = 0.0001). The 5-year overall survival (OS) for patients with higher and lower total MTV was 34.3% and 69.9% respectively (*p* < 0.0001). Multivariate analysis revealed, besides IPI, that total MTV was independently predictive for PFS (HR: 2.31, 95% CI: 1.16 – 4.60, *p* = 0.0180) and OS (HR: 2.38, 95% CI: 1.12 – 5.04, *p* = 0.024). TLG and maximal SUV of tumor were not independent prognostic factors.

**Conclusions:**

An elevated total MTV was a predictor for shorter PFS and OS in patients with DLBCL receiving rituximab-containing therapy, independent of IPI.

## INTRODUCTION

Diffuse large B-cell lymphoma (DLBCL), accounting for about one-third of all non-Hodgkin’s lymphoma (NHL), is the most common type of NHL [1]. The international prognostic index (IPI) had been a powerful prognostic tool for more than 20 years for stratifying patient risks [2]. The immuno-chemotherapy combining rituximab and cyclophosphamide, doxorubicin, vincristine, and prednisone (R-CHOP) has resulted in a significant improvement of survival [1]. However, a section of patients was not cured with R-CHOP, either due to primary refractory disease or late relapse following an initial response. Efforts have been made to improve the risk stratification model, including regrouping the IPI score (revised IPI) [3], initial hematological index [4], type of bone marrow involvement [5] and tumor bulk [6]. Nonetheless, these efforts have only resulted in an incremental improvement. New prognostic biomarkers for the rituximab era are needed.

Over the past decade, ^18^F-fluorodeoxyglucose (FDG) positron emission tomography, combined with computed tomography (PET/CT) has been widely used for the management of DLBCL [7-11]. The standardized uptake value (SUV) is the most commonly used semi-quantitative parameter in FDG PET/CT. Higher maximal SUV in the lesion has been proved to be of prognostic significance in patients with DLBCL [12, 13]. Beyond SUV, with the development of software programs, metabolic tumor volume (MTV) and total lesion glycolysis (TLG) have recently been found to play an important role in the prediction of patient outcomes. However, some recent studies evaluating the prognostic values of total MTV and TLG in DLBCL showed inconclusive and contradictory results [14-20].

Therefore, the aim of the current study was to determine the prognostic value of total MTV and TLG measured on pre-treatment FDG PET/CT, and to compare MTV and TLG with other clinical prognostic factors, in patients with newly diagnosed DLBCL receiving R-CHOP therapy.

## RESULTS

### Patient characteristics

A total of 118 patients, who met the inclusion criteria, were analyzed (Table 1). There were 63 (53.4%) men and 55 (46.6%) women, with mean age 61.8 ± 16.9 years at diagnosis. Forty-eight (40.7%) patients were at early stages (stage I or II), while the other 70 patients (59.3%) were at stages III or IV. According to IPI score, patients with low risk (0-1), low-intermediate risk (2), high-intermediate risk (3) and high risk (4-5) were 39, 36, 21 and 22 respectively. As for the revised IPI score, patients in “very good” prognostic group (score 0), “good” prognostic group (score 1-2) and “poor” prognostic group (score 3-5) were 11, 64 and 43 respectively. Seventy-one (60.2%) patients presented extranodal involvement and 20 (16.9%) patients presented with pathologically confirmed BM involvement at diagnosis.

**Table 1:** Baseline characteristics at diagnosis of the 118 patients with diffuse large B-cell lymphoma

Variable	Value (%)
Age (y)	
Mean ± SD	61.8 ± 16.9
Range	15-94
Sex	
Male	63 (53.4)
Female	55 (46.6)
Ann Arbor stage	
I	16 (13.6)
II	32 (27.1)
III	29 (24.6)
IV	41 (34.7)
IPI	
Low risk (score 0-1)	39 (33.1)
Low-intermediate (score 2)	36 (30.5)
High-intermediate (score 3)	21 (17.8)
High (score 4-5)	22 (18.6)
Revised IPI	
Very good (score 0)	11 (9.3)
Good (score 1-2)	64 (54.2)
Poor (score 3-5)	43 (36.4)
Primary lesions	
Lymph nodes	47 (39.8)
Extranodal lesions	71 (60.2)
Bone marrow involvement	
Yes	20 (16.9)
No	98 (83.1)

IPI: international prognostic index.

Baseline laboratory data including hemoglobin (Hb), white blood cell count (WBC), platelet count, albumin, creatinine, glutamate oxaloacetate transaminase (GOT), glutamate pyruvate transaminase (GPT), lactate dehydrogenase (LDH) and β2-microglobulin were collected. The imaging parameters acquired via FDG PET/CT scans were also measured (Table 2). The mean value of maximal SUV of tumor (SUVt) was 15.8 ± 8.2. The mean values of total MTV and TLG were 550.4 ± 678.3 cm^3^ and 3533.2 ± 4394.1 cm^3^ respectively.

**Table 2:** Baseline laboratory and imaging parameters of the 118 patients with diffuse large B-cell lymphoma

Variable	Mean (SD)
Hb (g/dL)	11.7 (1.9)
WBC (x 10^3^ μL)	6.9 (3.0)
Platelet (x 10^3^ /μL)	231.4 (103.2)
Albumin (g/dL)	3.6 (0.6)
Creatinine (mg/dL)	0.9 (0.7)
GOT (IU/L)	33.7 (24.0)
GPT (IU/L)	26.0 (17.2)
LDH (IU/L)	360.0 (503.0)
β2-microglobulin (μg/dL)	317.9 (287.2)
Maximal SUVt	15.8 (8.2)
MTV (cm^3^)	550.4 (678.3)
TLG (cm^3^)	3533.2 (4394.1)

SD: standard deviation; Hb: hemoglobin; WBC: white blood cell; GOT: glutamate oxaloacetate transaminase; GPT: glutamate pyruvate transaminase; LDH: lactate dehydrogenase; SUVt: standardized uptake value of tumor; MTV: metabolic tumor volume; TLG: total lesion glycolysis.

### Correlation between MTV, TLG and clinical prognostic parameters

Correlations between metabolic parameters from FDG PET/CT scans and clinical prognostic parameters are listed in Table 3. Using Spearman’s correlation test, total MTV was positively and significantly correlated with LDH level, creatinine level, GOT level, β2-microglobulin level, clinical stage, IPI score, revised IPI, bone marrow status, maximal SUVt and TLG. Inverse and significant correlations were seen between total MTV toward Hb and albumin level. On the other hand, TLG was positively and significantly correlated with LDH, GOT level, β2-microglobulin level, clinical stage, IPI score, revised IPI and maximal SUVt. Inverse and significant correlations were also seen between TLG toward Hb and albumin level.

**Table 3:** Correlations between metabolic parameters from FDG PET/CT scans and clinical prognostic parameters

	Total MTV		TLG	
	*r*	*p*	*r*	*p*
Hb	-0.275	0.0026^*^	-0.237	0.0097^*^
WBC	0.087	0.3482	0.089	0.3401
Platelet	0.065	0.4836	0.089	0.3368
Albumin	-0.488	<0.0001^*^	-0.453	<0.0001^*^
LDH	0.624	<0.0001^*^	0.577	<0.0001^*^
Creatinine	0.182	0.0484^*^	0.152	0.1003
GOT	0.369	<0.0001^*^	0.330	0.0003^*^
GPT	0.040	0.6691	0.025	0.7897
β2-microglobilin	0.491	<0.0001^*^	0.424	<0.0001^*^
Clinical Stage	0.467	<0.0001^*^	0.435	<0.0001^*^
IPI score	0.551	<0.0001^*^	0.475	<0.0001^*^
Revised IPI	0.557	<0.0001^*^	0.491	<0.0001^*^
Maximal SUVt	0.368	<0.0001^*^	0.533	<0.0001^*^
Total MTV	-	-	0.969	<0.0001^*^
TLG	0.969	<0.0001^*^	-	-

* statistically significant

MTV: metabolic tumor volume; TLG: total lesion glycolysis; *r*: correlation coefficients; Hb: hemoglolin; WBC: white blood cell; LDH: lactate dehydrogenase; GOT: glutamate oxaloacetate transaminase; GPT: glutamate pyruvate transaminase; SUVt: standardized uptake value of tumor; IPI: international prognostic index.

### Comparison between metabolic parameters measured in patients with different clinical outcomes

During the follow-up, patients who had progressive disease or had died, were grouped as progression (n = 55), as compared to patients in complete or partial remission (n = 63). After a median follow-up period of 28.7 months, 69 (58.5%) patients were alive and 49 (41.5%) patients had expired at the end of the study. The comparisons among maximal SUVt, total MTV and TLG in patients with different clinical outcomes was shown in Table 4. There were no significant differences in maximal SUVt between patients with progression and remission, and between patients who had expired and those who were alive. However, patients who underwent progression of disease had much higher total MTV and TLG, than patients with partial or complete remission (MTV, *p* = 0.0005; TLG, *p* = 0.0021). Patients who had expired had significantly higher total MTV and TLG, than patient who survived at the end of study (MTV, *p* < 0.0001; TLG, *p* = 0.0004).

**Table 4:** Comparisons between mean values of metabolic parameters measured in patients with different clinical outcomes

		Progression	Remission	*p*	Expired	Alive	*p*
		(n = 55)	(n = 63)		(n = 49)	(n = 69)	
Maximal SUVt	Mean	16.6	15.1	0.3288	16.0	15.7	0.7806
	SD	8.0	8.2		7.7	8.5	
Total MTV	Mean	745.1	380.5	0.0005^*^	828.1	353.2	<0.0001^*^
	SD	785.0	518.4		795.4	500.2	
TLG	Mean	4655.4	2553.6	0.0021^*^	5003.4	2489.2	0.0004^*^
	SD	5033.3	3504.1		4913.6	3676.4	

* statistically significant

SUVt: standardized uptake value of tumor; MTV: metabolic tumor volume; TLG: total lesion glycolysis; SD: standard deviation.

### Identification of the most discriminative cut-off values

**Figure 1:**
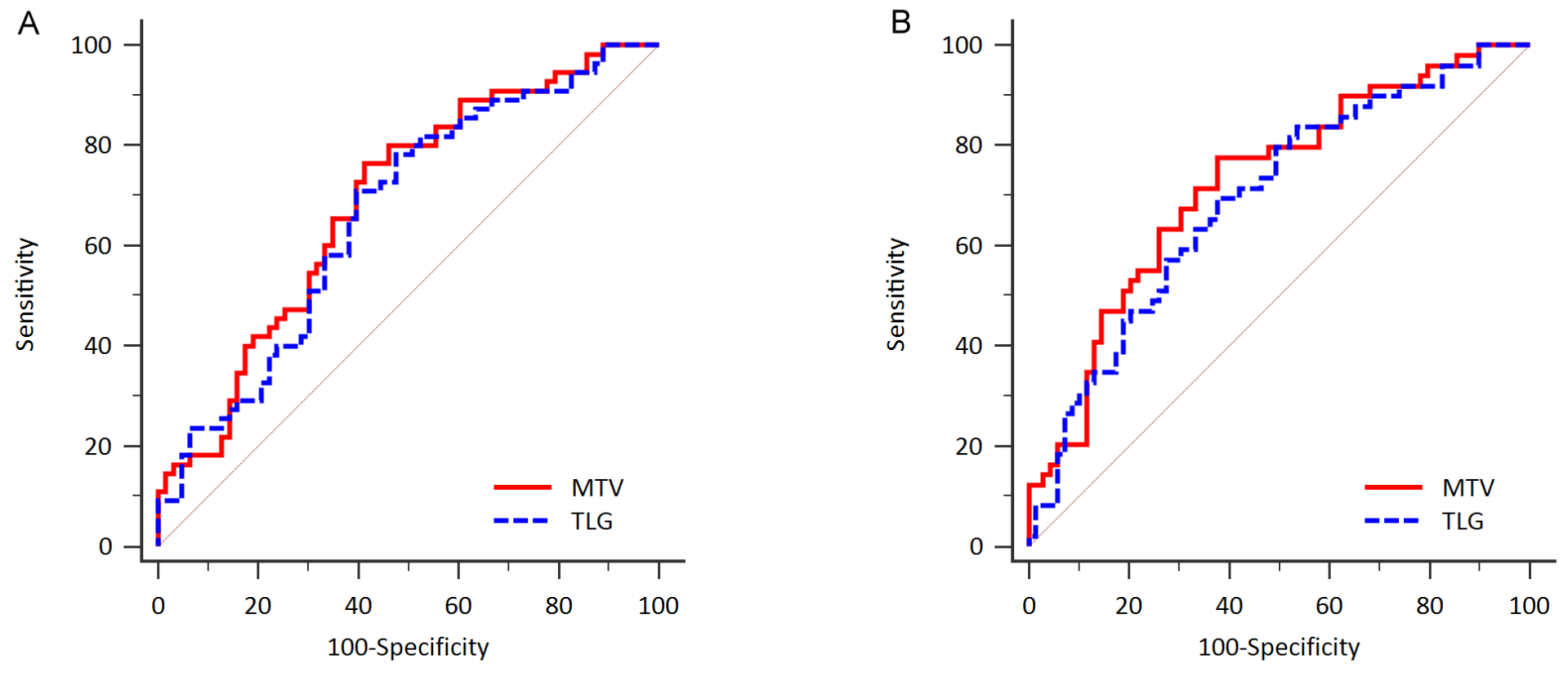
ROC curve analysis to determine the most discriminative cut-off value of total MTV and TLG **(A)** For prediction of PFS, AUCs were 0.687 for MTV (*p* = 0.0001) and 0.665 for TLG (*p* = 0.001). **(B)** For prediction of OS, AUCs were 0.723 for MTV (*p* < 0.0001) and 0.691 for TLG (*p* = 0.0001) respectively.

The receiver-operating characteristics (ROC) curve analysis was used to identify the ideal cut-off values in distinguishing high levels of MTV and TLG from low levels of MTV and TLG (Figure 1). For progression-free survival (PFS), the estimated areas under the ROC curve (AUCs) of MTV and TLG were 0.687 (*p* = 0.0001) and 0.665 (*p* = 0.001) respectively (Figure 1A). 165.4 cm^3^ was the best distinguishable cut-off value for dividing high and low MTV status, with 76.5% sensitivity and 58.7% specificity (Youden index 0.35). 1204.9 cm^3^ was the best determinative cut-off value for dividing high and low TLG status, with 70.9% sensitivity and 60.3% specificity (Youden index 0.31).

For overall survival (OS), the estimated AUCs of MTV and TLG were 0.723 (*p* < 0.0001) and 0.691 (*p* = 0.0001) respectively (Figure 1B). 190.2 cm^3^ was the best distinguishable cut-off value for dividing high and low MTV status, with 77.6% sensitivity and 62.3% specificity (Youden index 0.40). 1480.8 cm^3^ was the best determinative cut-off value for dividing high and low TLG status, with 69.4% sensitivity and 62.3% specificity (Youden index 0.32).

### Clinical outcomes according to cut-off values of MTV and TLG

**Figure 2:**
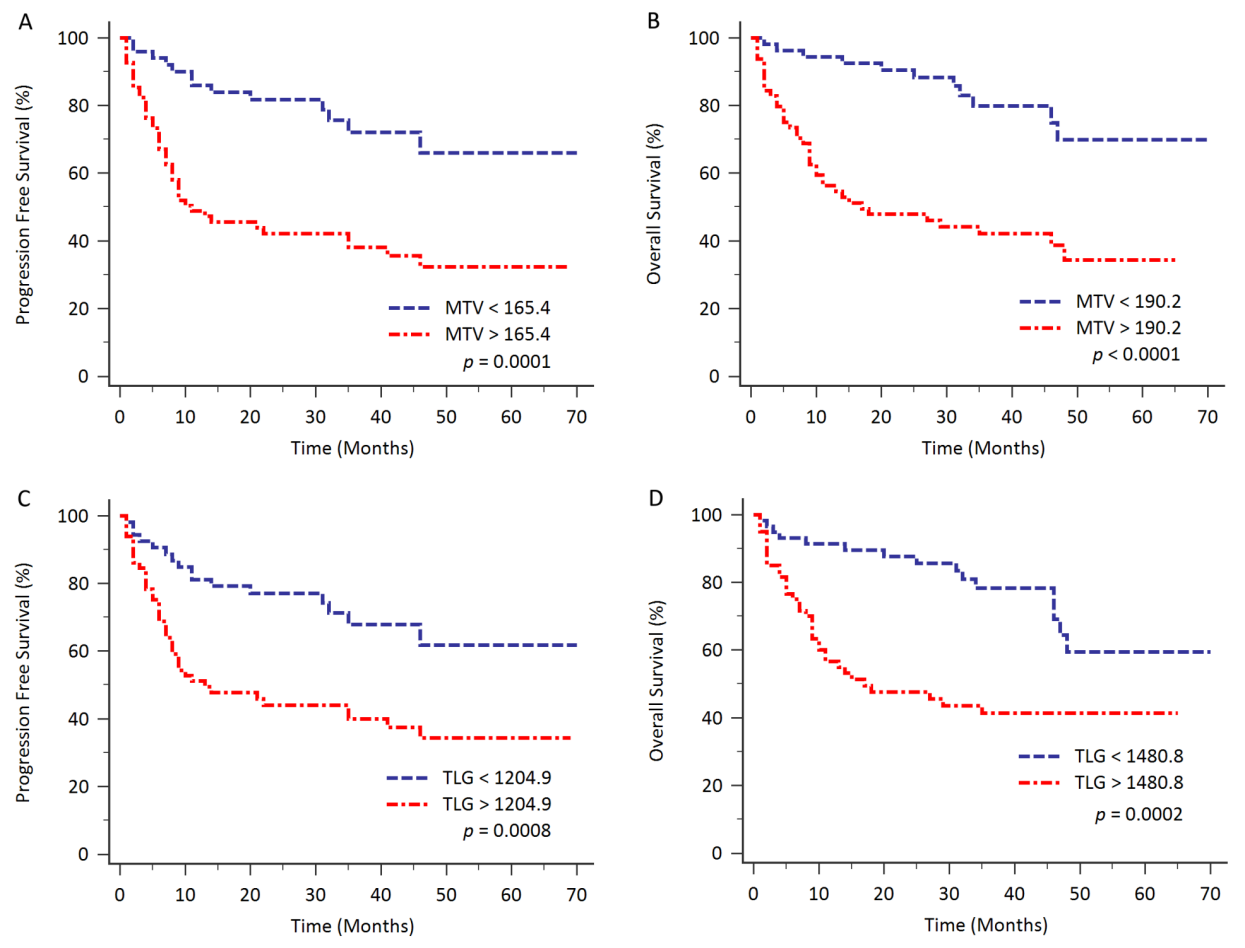
Kaplan-Meier analysis for evaluating the PFS and OS based on total MTV and TLG with different cut-off values Patients with higher total MTV had significantly shorter survival, compared to patients with lower total MTV (PFS, *p* = 0.0001; OS, *p* < 0.0001; Figure 2A and 2B). Patients with higher TLG also had significantly poorer outcome compared to patients with lower TLG (PFS, *p* = 0.0008; OS, *p* = 0.0002; Figure 2C and 2D).

In Kaplan-Meier survival analysis, patients with high MTV had poorer clinical survival, compared to patients with low MTV levels (PFS, cut-off value 165.4 cm^3^, *p* = 0.0001; OS, cut-off value 190.2 cm^3^, *p* < 0.0001; Figure 2A and 2B). The 5-year PFS for patients with high MTV (n = 68) and low MTV (n = 50) were 32.3% and 66.0% respectively. The 5-year OS for patients with high MTV (n = 64) and low MTV (n = 54) were 34.3% and 69.9% respectively. The median OS time for the patients with higher MTV (≥ 190.2 cm^3^, n = 64) was 17.0 months [95% CI: 10.0 – 48.0].

Similarly, patients with high TLG had inferior clinical survival, compared to patients with low TLG levels (PFS, cut-off value 1204.9 cm^3^, *p* = 0.0008; OS, cut-off value 1480.8 cm^3^, *p* = 0.0002; Figure 2C and 2D). The 5-year PFS for patients with high TLG (n = 65) and low TLG (n = 53) were 34.3% and 61.8% respectively. The 5-year OS for patients with high TLG (n = 60) and low TLG (n = 58) were 41.3% and 59.5% respectively. The median OS time for the patients with higher TLG (≥ 1480.8 cm^3^, n = 60) was also 17.0 months (95% CI: 10.0 – 35.0).

### Clinical outcomes in patients with different subgroups

Patients were divided into early-staged (staged I and II, n = 48) and late-staged (staged III and IV, n = 70) groups. In the early-staged group, patients with higher total MTV had poorer clinical outcomes (PFS, cut-off value 77.7 cm^3^, log-rank *p* = 0.0033; OS, cut-off value 77.7 cm^3^, log-rank *p* = 0.0193). Higher TLG also correlated with poorer clinical outcomes (PFS, cut-off value 475.6 cm^3^, log-rank *p* = 0.0095; OS, cut-off value 587.0 cm^3^, log-rank *p* = 0.0419).

In the late-staged group, patients with higher total MTV or TLG had poorer clinical PFS and OS. However, a significant difference of survival was only shown in the evaluation of OS using dichotomized total MTV (cut-off value 190.2 cm^3^, log-rank *p* = 0.0153).

### Comparison of clinical impacts of other prognostic parameters in all 118 patients

The Cox proportional hazard model was used to analyze the impact of MTV, TLG as well as clinical parameters on the clinical outcomes. The cut-off values of the laboratory data were dichotomized using normal reference, if available in the literature. The cut-off values of maximal SUVt, MTV and TLG were determined by the ROC curve analysis, as described in previous paragraph. For the evaluation of PFS, the univariate analysis revealed that lower platelet counts (< 172 × 10^3^/μL, *p* = 0.0084), lower albumin level (< 3.5 g/dL, *p* = 0.0236), higher LDH level (≥ 192 IU/L*, p* = 0.0006), higher total MTV (≥ 165.4 cm^3^, *p* < 0.0001), higher TLG (≥ 1204.9 cm^3^, *p* = 0.0009), bone marrow involvement (*p* = 0.0451), higher clinical stage (*p* = 0.0007), higher IPI score (*p* = 0.0001) and higher revised IPI score (*p* < 0.0001) were significantly associated with poorer clinical outcomes (Table 5). Further, multivariate analysis was conducted and revealed higher MTV [hazard ratio (HR): 2.31, 95% confidence interval (CI): 1.16 – 4.60, *p* = 0.0180], and high IPI score (high risk group, HR: 3.20, 95% CI: 1.42 – 7.18, *p* = 0.0050) had independent clinical impacts on PFS.

**Table 5:** Cox proportional hazards models analysis of potential prognostic factors affecting PFS

	Univariate analysis		Multivariate analysis	
	HR (95% CI)	*p*	HR (95% CI)	*p*
Sex (male vs. female)	1.22 (0.72-2.10)	0.4509		
Age (≥ vs.< 65 years)	1.44 (0.85-2.46)	0.1735		
Hemoglobin (< vs. ≥ 12.3 g/dL)	1.75 (0.98-3.10)	0.0501		
WBC (< vs. ≥ 4400 /μL)	1.31 (0.66-2.60)	0.4559		
Platelet (< vs. ≥ 172 × 10^3^/μL)	2.12 (1.24-3.65)	0.0084^*^		
Albumin (< vs. ≥ 3.5 g/dL)	1.87 (1.10-3.18)	0.0236^*^		
LDH (≥ vs. < 192 IU/L)	2.65 (1.48-4.77)	0.0006^*^		
Creatinine (≥ vs. < 1.3 mg/dL)	1.84 (0.93-3.65)	0.1037		
GOT (≥ vs.< 42 IU/L)	1.75 (0.98-3.14)	0.0703		
GPT(≥ vs.< 40 IU/L)	1.52 (0.80-2.89)	0.2210		
β2-microglobulin (≥ vs. < 340 μg/dL)	1.66 (0.92-2.99)	0.1042		
Maximal SUVt (≥ vs. < 18.8)	1.32 (0.77-2.24)	0.3154		
MTV (≥ vs. < 165.4 cm^3^)	3.32 (1.78-6.20)	<0.0001^*^	2.31 (1.16-4.60)	0.0180^*^
TLG (≥ vs. < 1204.9 cm^3^)	2.57 (1.43-4.61)	0.0009^*^		
BM invovlement (yes vs. no)	1.94 (1.05-3.57)	0.0451^*^		
Stage				
I	1	0.0007^*^		
II	1.60 (0.43-5.93)	0.4785		
III	4.15 (1.21-14.28)	0.0238^*^		
IV	4.99 (1.51-16.48)	0.0083^*^		
IPI				
Low (0-1)	1	0.0001^*^		0.0070^*^
Low-intermediate (2)	1.26 (0.57-2.75)	0.5697	0.98 (0.44-2.19)	0.9530
High-intermediate (3)	2.86 (1.30-6.31)	0.0087^*^	1.85 (0.79-4.30)	0.1550
High (4-5)	5.06 (2.40-10.68)	<0.0001^*^	3.20 (1.42-7.18)	0.0050^*^
Revised IPI				
1	1	<0.0001^*^		
2	1.43 (0.43-4.77)	0.5641		
3	4.60 (1.40-15.18)	0.0121^*^		

* statistically significant

HR: hazard ratio; CI: confidence interval; WBC: white blood cell; LDH: lactate dehydrogenase; GOT: glutamate oxaloacetate transaminase; GPT: glutamate pyruvate transaminase; SUVt: standardized uptake value of tumor; MTV: metabolic tumor volume; TLG: total lesion glycolysis; BM: bone marrow; IPI: international prognostic index

For the evaluation of OS, the univariate analysis disclosed that older age (≥ 65 years, *p* = 0.0032), lower Hb level (< 12.3 g/dL, *p* = 0.0010), lower platelet count (< 172 × 10^3^/μL, *p* = 0.0126), lower albumin level (< 3.5 g/dL, *p* = 0.0012), higher LDH level (≥ 192 IU/L, *p* = 0.0001), higher creatinine level (≥ 1.3 mg/dL, *p* = 0.0499), higher GOT level (≥ 42 IU/L, *p* = 0.0114), higher β2-microglobulin level (≥ 340 μg/dL, *p* = 0.0097), higher total MTV (≥ 190.2 cm^3^, *p* < 0.0001), higher TLG (≥ 1480.8 cm^3^, *p* = 0.0003), higher clinical stage (*p* = 0.0007), higher IPI score (*p* < 0.0001) and higher revised IPI score (*p* < 0.0001) were significantly associated with poorer clinical outcomes (Table 6). Further, multivariate analysis disclosed that older age (HR: 1.89, 95% CI: 1.03 – 3.48, *p* = 0.0410), higher total MTV (HR: 2.38, 95% CI: 1.12 – 5.04, *p* = 0.024) and higher IPI score (high-intermediate risk group, HR: 3.61, 95% CI: 1.18 – 11.02, *p* = 0.0240; high risk group, HR: 6.64, 95% CI: 2.17 – 20.32, *p* = 0.0010) had independent clinical impacts on OS.

**Table 6:** Cox proportional hazards models analysis of potential prognostic factors affecting OS

	Univariate analysis		Multivariate analysis	
	HR (95% CI)	*p*	HR (95% CI)	*p*
Sex (male vs. female)	1.03 (0.59-1.82)	0.9058		
Age (≥ vs.< 65 years)	2.35 (1.32-4.19)	0.0032^*^	1.89 (1.03-3.48)	0.0410^*^
Hemoglobin (< vs. ≥ 12.3 g/dL)	2.83 (1.44-5.55)	0.0010^*^		
WBC (< vs. ≥ 4400 /μL)	1.36 (0.66-2.81)	0.4179		
Platelet (< vs. ≥ 172 × 10^3^/μL)	2.12 (1.20-3.75)	0.0126^*^		
Albumin (< vs. ≥ 3.5 g/dL)	2.56 (1.46-4.49)	0.0012^*^		
LDH (≥ vs. < 192 IU/L)	3.30 (1.72-6.35)	0.0001^*^		
Creatinine (≥ vs. < 1.3 mg/dL)	2.12 (1.06-4.25)	0.0499^*^		
GOT (≥ vs.< 42 IU/L)	2.23 (1.23-4.02)	0.0114^*^		
GPT(≥ vs.< 40 IU/L)	1.60 (0.81-3.13)	0.1933		
β2-microglobulin (≥ vs. < 340 μg/dL)	2.28 (1.26-4.12)	0.0097^*^		
Maximal SUVt (≥ vs. < 18.8)	1.22 (0.69-2.14)	0.5022		
MTV (≥ vs. < 190.2 cm^3^)	4.05 (2.07-7.95)	<0.0001^*^	2.38 (1.12-5.04)	0.0240^*^
TLG (≥ vs. < 1480.8 cm^3^)	2.96 (1.61-5.45)	0.0003^*^		
BM invovlement (yes vs. no)	1.33 (0.66-2.69)	0.4332		
Stage				
I	1	0.0007^*^		
II	1.01 (0.25-4.04)	0.9889		
III	4.00 (1.17-13.67)	0.0270^*^		
IV	3.84 (1.15-12.81)	0.0285^*^		
IPI				
Low (0-1)	1	<0.0001^*^		0.0030^*^
Low-intermediate (2)	3.14 (1.12-8.81)	0.0301^*^	2.21 (0.76-6.45)	0.1440
High-intermediate (3)	6.23 (2.19-17.73)	0.0006^*^	3.61 (1.18-11.02)	0.0240^*^
High (4-5)	14.2 (5.27-38.36)	<0.0001^*^	6.64 (2.17-20.32)	0.0010^*^
Revised IPI				
1	1	<0.0001^*^		
2	3.36 (0.45-25.27)	0.2391		
3	14.3 (1.94-104.7)	0.0090^*^		

* statistically significant

HR: hazard ratio; CI: confidence interval; WBC: white blood cell; LDH: lactate dehydrogenase; GOT: glutamate oxaloacetate transaminase; GPT: glutamate pyruvate transaminase; SUVt: standardized uptake value of tumor; MTV: metabolic tumor volume; TLG: total lesion glycolysis; BM: bone marrow; IPI: international prognostic index

## DISCUSSION

FDG PET/CT scan has been widely used in the oncological field for several years. The clinical roles of FDG PET/CT scan in diagnosis, staging, monitoring of treatment and prediction of prognosis in patients with lymphoma have been reported [7-11]. The maximal SUV of the primary tumor has been previously demonstrated to be of prognostic values, because of easy accessibility and high reproducibility [12, 13]. However, maximal SUV solely recorded intensity of FDG uptake in the most aggressive cells, without reflecting the volumetric concept. The volumetric analysis of MTV and TLG, providing more information than maximal SUV, has brought increasing evidences of clinical value. Meignan *et al.* collected pooled data from three clinical trials dealing with follicular lymphoma, and found that higher MTV yielded poor clinical outcomes based on PFS [21]. Cottereau *et al.* reported that higher MTV predicted a poor survival in patients with peripheral T-cell lymphoma [22]. Kanoun *et al.* [23] and Ceriani *et al.* [24] had similar reports in Hodgkin’s lymphoma and primary mediastinal (thymic) large B-cell lymphoma respectively.

In DLBCL, Song *et al.* conducted a retrospective analysis on 169 patients with nodal stage II and III DLBCL, in which MTV had more potential predictive power than Ann Arbor stage [25]. Sasanelli *et al.* had similar results suggesting that pre-therapy total MTV is an independent predictor of outcome in all staged patients [17]. In patients with bone marrow involvement, it was concluded by Song *et al.* that high total MTV predicted worse prognosis [26]. Another article by Song and his colleagues concluded that high MTV is an independent factor for predicting survival in primary gastrointestinal DLBCL [27]. Combining early PET/CT response or molecular characteristics, MTV also improved the predictive power and defined a poor prognosis group [20], and made accurate selection of patients to increase tailored therapy [28]. However, conflicting results coexisted. Some articles mentioned that TLG, but not MTV, was the better predictor and correlated well with the patient outcomes [15, 16, 18]. Some articles presented that neither total MTV nor TLG on FDG PET/CT scan was independent predictor [14, 19, 29].

In the current study, we have demonstrated that both total MTV and TLG had the clinical potential to predict PFS and OS in patients with DLBCL receiving R-CHOP chemotherapy. Total MTV and TLG were significantly correlated with hematological (e.g. Hb, albumin, LDH, creatinine, GOT and β2-microglobulin) and clinical (stage, IPI score, revised IPI score and maximal SUVt) parameters. Furthermore, total MTV and IPI score were the only two independent prognostic factors to predict poor PFS and OS. Using the cut-off value determined by ROC curves (PFS, 165.4 cm^3^; OS, 190.2 cm^3^), patients with higher MTV had a poorer 5-year PFS and OS (PFS, 32.3% vs. 66.0%; OS, 34.3% vs. 69.9%) respectively. Our result is similar to that reached in earlier articles by Song and Sasanelli *et al.* [17, 25-27], in spite of different patient populations. In the current study, TLG was statistically significant in univariate analysis, but failed to be an independent factor in multivariate analysis. We speculated that the cause may be related to different definitions of marginal threshold, when measuring the MTV and mean SUV of lesion. Most studies, in which MTV were more predictive for patient outcomes, used absolute cut-off of SUV (more than 2.5), as the threshold to define MTV [20, 25-27], while only one article used 41% threshold of maximal SUV to calculate MTV [17]. The similarities and differences between the current study and previous similar studies in DLBCL were summarized in Table 7.

**Table 7:** Studies on the prognostic values of MTV and TLG in DLBCL

Study	Patient numbers	Treatment	MTV (cm^3^)		TLG (cm^3^)		Prognostic significance
			Median	range	Median	range	
Adams et al [14]	73	R-CHOP	445	6-2454	4898	13-23322	Neither MTV nor TLG predicted outcome.
Esfahani et al [15]	20	R-CHOP	NA	NA	NA	NA	TLG 705 cm^3^ yielded PFS 56.5 vs. 29.2 months.
Kim et al [16]	140	R-CHOP	NA	NA	416	5-1499	TLG 416 cm^3^ yielded 2-year PFS 92% vs.73%.
Sasanelli et al [17]	114	R-CHOP or ASCT	315	4-2654	2974	14-21908	MTV 550 cm^3^ yielded 3-year OS 87% vs. 60%.
Zhou et al [18]	91	R-CHOP	51	IQR: 17-151	497	IQR: 104-1452	TLG 827 cm^3^ yielded 5-year PFS 83% vs. 34%; 726 cm^3^ yielded 5-year OS 92% vs. 67%.
Gallicchio et al [19]	52^*^	R-CHOP or R-COMP	43	2-340	597	110-2552	SUVmax rather than MTV and TLG remained the only predictor for EFS.
Mikhaeel et al [20]	147	R-CHOP	595	2-7357	4670	6-36570	MTV combined i-PET Deauville score had most predictive power.
Song et al [25]	169^+^	R-CHOP	198	5-1991	NA	NA	MTV 220 cm^3^ yielded 5-year PFS 90% vs. 56%; 5-year OS 93% vs. 58%.
Song et al [26]	107^#^	R-CHOP	527	15-3549	NA	NA	Total MTV 601 cm^3^ yielded significance difference in both PFS and OS.
Song et al [27]	165^※^	R-CHOP or Surgery + R-CHOP	133	10-654	NA	NA	High IPI, High MTV, and surgical resection followed by R-CHOP were independent prognostic factors for PFS and OS.
Cottereau et al [28]	81	R-CHOP R-ACVBP	320	IQR: 106-668	3677	IQR: 1066-6096	MTV 300 cm^3^ yielded 5-year PFS 76% vs. 43%; 5-year OS 78% vs. 46%.
The current study	118	R-CHOP	249	2-2970	1531	3-18106	MTV 165 cm^3^ yielded 5-year PFS 66% vs. 32%; 190 cm^3^ yielded 5-year OS 70% vs. 34%.

Abbreviations:

MTV: metabolic tumor volume; TLG: total lesion glycolysis; R-CHOP: rituximab, cyclophosphamide, doxorubicin, vincristine, and prednisone; NA: not available; PFS: progression-free survival; ASCT: autologous stem cell transplantation; OS: overall survival; IQR: interquartile range; R-COMP: rituximab, cyclophosphamide, hydroxydaunorubicin liposomal, vincristine, prednisone; SUVmax: maximal standardized uptake value; EFS: event-free survival; i-PET: interim positron emission tomography; IPI: international prognostic index; R-ACVBP: rituximab, doxorubicine, vindesine, cyclophosphamide, bleomycin, prednisolone.

* In patients with intermediate IPI score

^+^ In staged II and III patients without extranodal site involvement

^#^ In patients with bone marrow involvement of lymphoma

^※^ In patients with stage IE or IIE primary gastrointestinal DLBCL

In the literature review, we found that total MTV and TLG differed in a wide range among earlier reports. The reasons may be related to patient characteristics with a wide range of age, clinical stage and different subtypes of disease. Another important reason was related to the different software and the different ways used to define the marginal threshold with abnormal FDG uptake. There were multiple programs provided by different vendors used to calculate the MTV, e.g. Syngo TrueD (Siemens Healthcare) [14, 15], Planet Onco (DOSISoft) [28], PET-VCAR program (GE Healthcare) [21, 29], Imagys (Keosys, Saint-Herblain, France) [17, 21] and so on. There was a paucity of inter-program correlations and discrepancy.

As to the methodology, there are three basic methods to evaluate the MTV. The first one is according to the threshold percentage of maximal SUV in a lesion [30]. Some authors adopted this method with different thresholds, ranging from 40% to 42% [14, 17, 19, 28, 29]. One article compared 3 settings of marginal thresholds (i.e. 25%, 50% and 75%) to get an optimal one [16]. We think that there are drawbacks in using this methodology. If the maximal SUV of lesion is relatively high, the metabolic volume will be underestimated. For example, if we use a threshold of 40% to estimate the volume of a lesion with maximal SUV of 18, the portion with SUV below 7.2 will not be included in the further calculation. That is the reason why the ideal threshold should be different according to maximal SUV, in the earlier articles. The second method to define threshold is according to the mean SUV of normal liver plus 3 standard deviations (SD) [18, 31]. This method is patient-based and is able to reduce the influence of different PET/CT system and technical or artificial factors. However, the mean SUV of normal liver should be carefully defined, especially in patients who presented with hepatic involvement by lymphoma at the diagnosis. In the current study, we used the third method, in which lesions with an absolute cut-off value of SUV more than 2.5 were incorporated into calculation of total MTV, as suggested by Freudenberg *et al.* [32]. The method was also adopted in several articles [20, 25-27]. The important things regarding this method are to control the imaging protocols, including patient preparation, as consistently as possible. Under the reading of experienced nuclear physicians, the advantage of this method is that it is easy to define the lesion with a clear-cut value. Several other methods, such as gradient-based, statistical- and texture-based methods for auto-segmentation of PET volumes exist. Every method has its specific advantages and disadvantages. To the best of our knowledge, there is no published technical standard to confirm complete accuracy in measuring the metabolic volumes in all organs and settings. However, a normalized and standardized method to calculate the metabolic volume is necessary, because baseline metabolic tumor volume values were significantly influenced by the choice of the method used for determination of volume [33].

In the dichotomization of ideal cut-off values of total MTV and TLG, most articles used a retrospective ROC analysis to determine the optimal values. Only one article used X-tile analysis to determine the value [21]. Some authors didn’t mention the dichotomizing method in their articles [14, 29]. More reliable analytic tests have been provided. X-tile is a graphical method that illustrates the presence of substantial tumor sub-populations and shows the relationship between a biomarker and outcome by construction of a two dimensional projection of every possible subpopulation [34]. The time-dependent ROC curve is another method, which allows for time-varying marker effects and accommodates censored failure time outcome [35, 36]. Further validations with more sophisticated analytic tests may be necessarily applied.

Although the current study was relatively small with a retrospective design, the results underlined the prediction of poor PFS and OS in DLBCL patients with higher total MTV on the pre-treatment FDG PET/CT scan. In addition to the IPI score, the higher total MTV helped to identify the high-risk patients. Early identification of high-risk patients allowed clinicians to pay more attention to the treatment strategies and follow-up [37]. Further prospective study with a larger patient population and a more specific histological subtype collection may be conducted.

## CONCLUSION

Our study indicated that total MTV on pre-treatment FDG PET/CT scans was an independent predictor for survival in patients with DLBCL receiving R-CHOP therapy. An elevated total MTV was associated with poorer PFS and OS.

## MATERIALS AND METHODS

### Patient population

We performed a retrospective analysis of patients with DLBCL who were diagnosed between September 2009 and December 2014 and received treatment in Kaohsiung Medical University Hospital. Patient consent was waived because all the clinical data were retrospectively collected via medical chart review. However, informed consent before every examination including FDG PET/CT scan was required. The inclusion criteria for this study were as follows: (a) the diagnosis of DLBCL was pathologically proved, (b) complete pre-treatment work-up including history, physical examination, standard laboratory tests, as well as bone marrow aspiration and biopsy were available, (c) a whole-body FDG PET/CT scan was performed for pre-treatment staging, (d) first-line treatment with 6 or 8 cycles of R-CHOP therapy. The exclusion criteria were a previously known history of other malignance and the central nervous involvement of DLBCL. Patients were staged clinically with Ann Arbor staging criteria. The study design and review process was approved by the Institutional Review Board of Kaohsiung Medical University Hospital.

### FDG PET/CT acquisition

All the FDG PET/CT images were acquired using the Discovery ST 16 PET/CT scanner (GE Medical System, Waukesha, Wisconsin, USA). Every patient was asked to fast for at least 6 hours prior to the examination. The blood glucose level was measured to enssure no more than 150 mg/dl before the tracer injection. After intravenous injection of 370-555 MBq (10-15 mCi) of ^18^F-FDG, patients were asked to lie comfortably to reduce muscular uptake. The mean uptake time was 55 ± 5 minutes. Spiral low dose CT scan (140 kV, 80 mA, 3.75 mm section thickness) was acquired with a craniocaudal direction and an “arm up” position, followed by the emission acquisition with a reverse direction. The emission scan time per bed was 4 minutes. PET images were reconstructed iteratively (order subset expectation maximization) with CT data for attenuation correction. The Xeleris Functional Imaging Workstation (GE Medical System, Waukesha, Wisconsin, USA) was used for image display and interpretation.

### FDG PET/CT analysis

The image interpretation and SUV measurement were performed by two nuclear medicine physicians, who were blinded to the patients’ clinical outcomes. A positive lesion on PET/CT was defined as focal or diffuse FDG uptake above the background and was not compatible with a physiological normal uptake [38]. Disagreements were resolved by discussion to reach a consensus interpretation. Using CT images from the FDG PET/CT, the maximal SUVt was collected by drawing a region of interest (ROI) over the most intense slice of the primary lesions. The MTV was defined as the volume of hyper-metabolic lesion, with an SUV greater than a threshold of 2.5, as previous literature suggested [32]. To measure MTV values, PET/CT data were transferred in DICOM format to an OsiriX workstation (OsiriX MD 8.0, Pixmeo Sari, Bernex, Switzerland). Using the 3-dimensional segmentation, a 3-dimensional ROI as well as the contour including each hyper-metabolic lesion previously recognized was automatically produced. The voxels presenting SUV values more than 2.5 within the contour margin were then incorporated, in order to calculate the tumor volumes. The mean SUV of the delineated volume was also provided, using the in-house SUV-based automated contouring program. The total MTV of each patient was defined as the summation of MTVs of all focal lesions selected. The TLG was obtained by multiplying the MTV of every focal lesion by the corresponding mean SUV. The whole-body TLG of each patient was determined by the summation of the TLGs of all focal lesions selected.

### Treatment and clinical course

PFS was defined as the time from diagnosis to disease relapse, progression or death. OS was defined as the time from diagnosis to death from any cause. All patients received 6 or 8 cycles of R-CHOP for the initial therapy. Involved field radiation therapy was administered for clinically indicated patients, i.e. initial bulky disease (≥ 10 cm) or residual tumor presented, after completion of chemotherapy. Complete remission (CR) was defined by follow-up image evaluation, either by FDG PET/CT or CT scan, according to published criteria [38]. Patients with refractory and relapsed disease were treated with salvage chemotherapy or received autologous stem cell transplantation (ASCT) with high-dose chemotherapy, if clinically indicated. The observation period was from September 2009 to January 2016.

### Statistical analysis

Continuous variables were presented as mean (SD) and categorical data were given as frequencies (percentages). A Kolmogorov-Smirnov test was used to determine whether the variable was of normal distribution or not. The Spearman’s rank correlation test was used to analyze the correlation between metabolic parameters from FDG PET/CT and clinical prognostic factors. The Mann-Whitney test was conducted to compare metabolic parameters measured in patients with different clinical outcomes. The optimal cut-off values for total MTV and TLG were determined by ROC curves analysis. The survival curves were obtained by the Kaplan-Meier analysis in the groups dichotomized by optimal cut-off values of metabolic parameters. The survival difference between groups was evaluated by the log-rank test. A Cox proportional hazard model with univariate and multivariate analysis was conducted, to evaluate the impact of every clinical and metabolic parameter on patient survival. The HR and its 95% CI, calculated by Cox proportional hazard model were presented. All these analyses were performed using MedCalc Statistical Software version 17.4.4 (MedCalc Software bvba, Ostend, Belgium; http://www.medcalc.org; 2017). All statistical tests were two-sided, and a two-tailed *p* < 0.05 was considered significant.
